# Association of leukocyte telomere length and the risk of age-related hearing impairment in Chinese Hans

**DOI:** 10.1038/s41598-017-10680-9

**Published:** 2017-08-31

**Authors:** Han Liu, Huajie Luo, Tao Yang, Hao Wu, Dan Chen

**Affiliations:** 1 Ministry of Education and Shanghai Key Laboratory of Children’s Environmental Health, Xin Hua Hospital Affiliated to Shanghai Jiao Tong University School of Medicine, Shanghai, China; 2 Department of Otolaryngology, Renji Hospital Affiliated to Shanghai Jiao Tong University School of Medicine, Shanghai, China; 3 Department of Otolaryngology–Head and Neck Surgery, Xinhua Hospital Affiliated to Shanghai Jiao Tong University School of Medicine, Shanghai, China; 4 Department of Otorhinolaryngology-Head and Neck Surgery, Shanghai Ninth People’s Hospital Affiliated to Shanghai Jiaotong University School of Medicine, Shanghai, China; 5 Ear Institute, Shanghai Jiaotong University School of Medicine, Shanghai, China; 6 Shanghai Key Laboratory of Translational Medicine on Ear and Nose Diseases, Shanghai, China

## Abstract

Age-related hearing loss (ARHI) is the most common sensory disorder in the elderly. Although telomere attrition has been shown as a determinant in the pathobiology of various age-related diseases, it remains unknown whether telomere length is associated with ARHI. We hypothesized that decreased leukocyte telomere length (LTL) increased the risk of ARHI. Thus, we measured LTL of 666 ARHI and 43 controls by an established quantitative PCR technique. Four audiogram shape subtypes of ARHI, including “flat shape (FL)”, “2–4 kHz abrupt loss (AL) shape”, “8 kHz dip (8D) shape” and “sloping shape (SL)” could be identified among the cases using K-means cluster analysis. Longer LTL was associated with the reduced incidence of ARHI (adjusted OR = 0.550, 95% CI: 0.420–0.721, *P* < 0.0001 for all the ARHI; 0.498, 0.318–0.780, *P* = 0.0023 for FL subgroup; 0.428, 0.292–0.628, *P* < 0.0001 for AL subgroup; 0.552, 0.399–0.764, *P* = 0.0003 for mSL subgroup). Subjects in the highest tertile of LTL were at less risk for ARHI than those in the lowest and middle tertiles (OR for ARHI: 0.327, 95% CI 0.170–0.629, *P* = 0.0008). There was a descending trend of LTL as the degree of pure tone threshold average (PTA) aggravated. These results suggest that telomere attrition may be involved in the progression of ARHI.

## Introduction

Age-related hearing loss (ARHI), also known as presbycusis, is defined as a bilateral progressive high-frequency hearing loss that is demonstrated on an audiometric assessment by a pure tone audiogram^1^. It is the most common sensorineural impairment among adults aged older than 50. Prevalence of ARHI in China is on a rapid increase due to the population aging. ARHI leads to auditory deterioration and communication difficulties which aggravate the unhealthy psychological status of the patients and reduce the quality of life for the elderly. Therefore, it is important to elucidate the influencing factors of ARHI and prevent the progression of it. Studies have shown that multiple genetic and environmental factors interacted during the development of ARHI, including genetic mutations such as mitochondrial DNA deletions^2^, altered vascular characteristics such as increased vascular permeability and excess production of reactive oxygen species (ROS)^3^. In current literature, free radical or mitochondrial clock theory of aging is the most intriguing among the various mechanisms that are postulated to participate in the course of ARHI. Hypoperfusion of the cochlear tissue triggered by a reduction in blood flow with aging leads to ischemia and formation of ROS. Accumulated free radical damage over time results in mitochondrial DNA deletions which may be associated with aging and presbycusis.

Telomeres consist of repetitive DNA sequence TTAGGG extending from a few to 15 kilobases in length and some specific bound proteins at the end of the eukaryotic chromosomes^4^. They are essential for maintaining successful DNA replication and chromosomal integrity. Telomeres shorten by 30–200 base pairs with each division in somatic human cells and result in cell senescence when reach to Hyflick limit^5^. Evidence suggests that in most cell strains, it is oxidative damage that is principally responsible for the telomere loss by accumulation of single strand breaks^6^. During the process of oxidative stress, telomere is shortening at a faster speed and assessment of telomere length might be a significant indicator of disease progression. Accelerated telomere shortening leads to chromosomal instability which further increases the risk of genetic mutations and chromosome abnormalities. Since first reported by Harley *et al*.^7^ in 1990, increasing evidence indicates that, as a biomarker of cell senescence and oxidative stress, telomere shortening with aging is a determinant in the pathobiology of human age-related diseases such as cardiovascular disease^8^, type 2 diabetes^9^, and earlier mortality^10^ etc. Therefore, there is a hypothesis that changes in telomeres may predispose to the development of ARHI^11^ and it is possible that oxidative stress induced DNA damage in patients with ARHI could translate into accelerated telomere loss and a progression to the senescence of the hearing cells.

Until now, there has been no research to test the relationship between telomere length and ARHI. Since oxidative stress has a close association with both telomere length and ARHI, we hypothsized that ARHI patients would demonstrate shorter telomeres compared with controls. Hence, in our study, we measured mean leukocyte telomere length (LTL) in a large sampe size of ARHI patients and controls to analyze the role of telomere length in the pathophysiology of ARHI.

## Results

**Figure 1:**
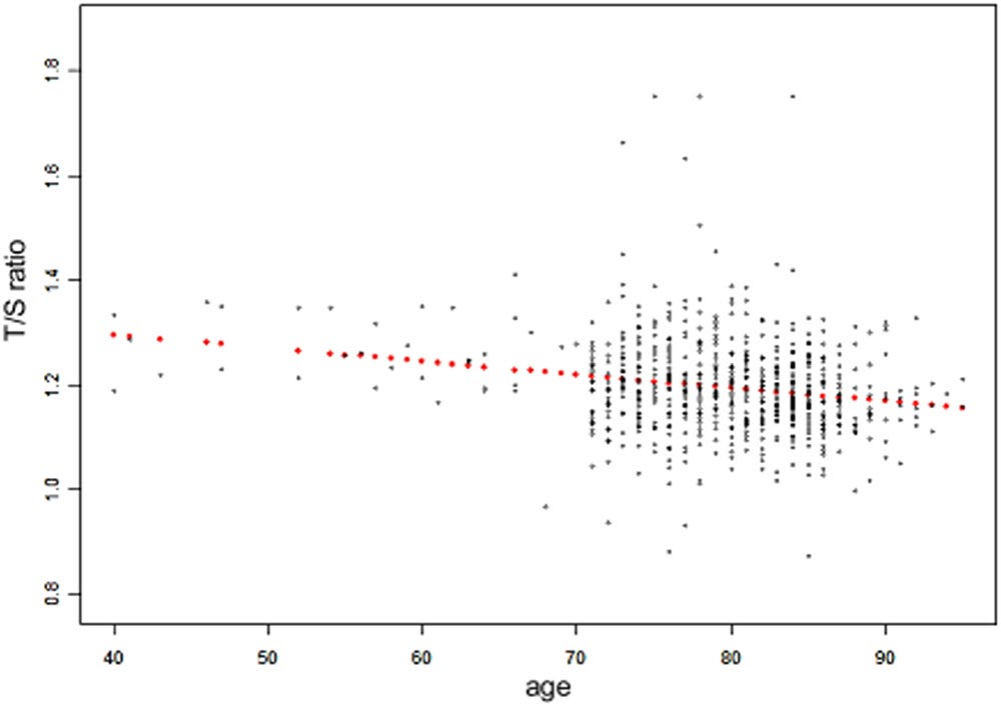
The correlation coefficient (r) between LTL and age was −0.146 (*P* < 0.0001) for all the subjects (N = 709). Linear regression line was drawn in red points.

DNA samples of 666 ARHI cases and 43 controls were available in this study. The characteristics of the study subjects is shown in Table 1. Average age of the ARHI cases and controls were 80.00 ± 5.50 (range from 70 to 95) years and 75.19 ± 3.89 (range from 70 to 85) years, respectively. Mean pure tone threshold average (PTA) in the better ear was 43.39 ± 13.73 dB HL and 21.69 ± 1.89 dB HL at 0.5, 1, 2, and 4 kHz for the cases and controls, respectively. None of the variables except age was associated with both ARHI and LTL. Therefore, we adjusted for age in the subsequent analysis. T/S ratio (telomere/single copy gene-ratio) of LTL and age were normally distributed in this study assessed by Kolmogorov–Smirnov tests. As presented in Fig. 1, LTL declined with age increasing. Relationship between LTL and age for all participants that was deduced by linear regression analysis is: T/S ratios of LTL = −0.002 (age in years) +1.388 (correlation coefficient [r] = −0.146 by Pearson correlation analysis; P < 0.0001).

**Table 1:** Characteristics of ARHI patients and controls.

Characteristic	Total (N = 709)	ARHL		*P* Value
		Yes (N = 666)	No (N = 43)	
Age, y	79.70 ± 5.54	80.00 ± 5.50	75.19 ± 3.89	<0.0001
PTA, dB HL	42.07 ± 14.29	43.39 ± 13.73	21.69 ± 1.89	<0.0001
Central Obesity	434(61.2)	413(62.0)	21(48.8)	0.443
Coronary heart disease	196(27.6)	192(28.8)	4(9.3)	0.015
Hypertension	188(26.5)	172(25.8)	16(37.2)	0.023
Diabetes mellitus	135(19.0)	125(18.8)	10(23.3)	0.231
Dyslipidemia	427(60.2)	418(62.8)	9(20.9)	<0.0001
Smoking	664(93.7)	637(95.6)	27(62.8)	<0.0001
Drinking	635(89.6)	607(91.1)	28(65.1)	<0.0001

Missing data in ARHI patients equals to 27. Missing data in controls equals to 7. Groups difference of every variable were compared using t tests or X^2^ tests where appropriate.

**Figure 2:**
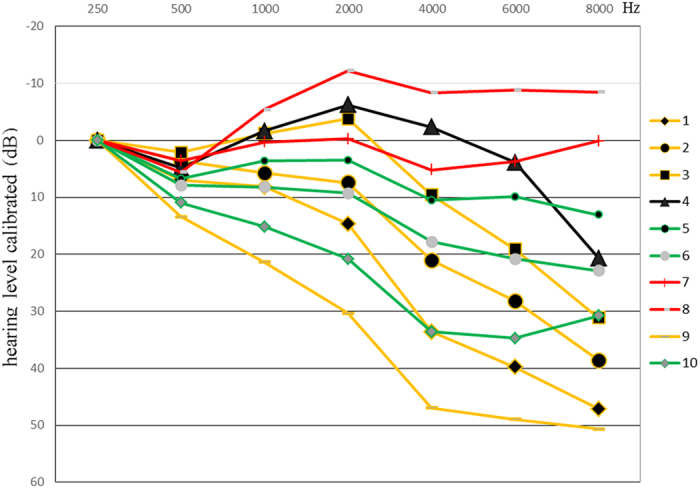
Illustrates shape 10- solutions of ARHI group audiogram patterns classified by k-means cluter analysis. For facilitating comparison, all audiometric shapes are calibrated at the 250 Hz frequency. ARHI cases can be divided into four subgroups: the FL shape (red line 7, 8), the 8 kHz dip (8D) shape (black line 4), the 2–4 kHz AL shape (yellow line 1, 2, 3, 9) and the mSL shape (green line 5, 6, 10).

F statistic and mean square errors obtained by ANOVA analysis were used to determine the ideal number of audiogram shapes as 10. According to the AMCLASS^TM^ standard description^12^, the 10 audiogram shapes could be described as follows based on the K-means cluster analysis: A flat (FL) shape (N = 92), An 8 kHz dip (8D) shape (N = 36), A 2–4 kHz abrupt loss (AL) shape (N = 305) and A mild sloping (mSL) shape (N = 233) (Table 2). As illustrated in Fig. 2, A FL shape is the audiogram where all octave thresholds fluctuate within ±10 dB difference to the mean (red line 7, 8). An 8D shape is the audiogram where the 8 kHz threshold is worsen than others by more than 20 dB (black line 4). A 2–4 kHz AL shape is the audiogram where a difference of more than 15 dB exists between 2 kHz and 4 kHz (yellow line 1, 2, 3, 9). A mSL shape is the audiogram where higher frequency thresholds are poorer than lower frequency thresholds by 10–40 dB (green line 5, 6, 10). In the logistic regression analysis between ARHI patients and controls, decreased T/S ratio was associated with the incidence of ARHI (adjusted OR = 0.550, 95% CI: 0.420–0.721, *P* < 0.0001 for all the ARHI patients; 0.498, 0.318–0.780, *P* = 0.0023 for FL subgroup; 0.428, 0.292–0.628, *P* < 0.0001 for AL subgroup; 0.552, 0.399–0.764, *P* = 0.0003 for mSL subgroup; Table 3). However, the OR between 8D subgroup of ARHI cases and controls failed to reach statistical significance.

**Table 2:** Nomenclature and subgroups of audiogram 10 shapes.

Nom of configuration	Audiogram line number	N(%)	Total N(%)
Flat shape (FL)	7	56(8.41%)	92(13.81%)
	8	36(5.41%)	
8 kHz dip (8D) shape (8D)	4	36(5.41%)	36(5.41%)
2–4 kHz abrupt loss shape (AL)	1	60(9.01%)	305(45.80%)
	2	93(13.96%)	
	3	79(11.86%)	
	9	73(10.96%)	
Mild sloping shape (mSL)	5	74(11.11%)	233(34.98%)
	6	102(15.31%)	
	10	57(8.56%)	
Total in case group			666(100%)

**Table 3:** Association between T/S ratio and the presence of ARHI or its subgroups.

	ARHI Patients					Controls (N = 43)
	(N = 666)					
	Total	FL(N = 92)	8D(N = 36)	AL(N = 305)	SL(N = 233)	
T/S ratio	1.190 ± 0.087	1.188 ± 0.098	1.213 ± 0.088	1.190 ± 0.080	1.188 ± 0.092	1.270 ± 0.126
OR adjusted for age	0.550	0.498	0.641	0.428	0.552	
95% CI	0.420–0.721	0.318–0.780	0.372–1.107	0.292–0.628	0.399–0.764	
*P*	<0.0001	0.0023	0.1105	<0.0001	0.0003	

ARHI cases could be divided into four subgroups according to K-means cluster analysis. T/S ratio was shown as mean ± SD for each group. OR (odds ratio), 95% CI (Confidence Interval) and *P* value indicated the association between T/S ratio of LTL and the presence of ARHI by the use of an age adjusted logistic regression model.

Based on the standards recommended by the World Health Organization in 2005, the degrees of hearing impairment were classified as: normal hearing: PTA lower than 25 dBHL; mild hearing impairment: 26–40 dBHL; moderate hearing impairment: 41–70 dBHL; severe and extremely severe hearing impairment: >71 dBHL. As presented in Table 4, there was a descending trend of LTL as the degree of hearing impairment aggravated after adjusting for age (*P* = 0.03).

**Table 4:** Trend of LTL according to the degree of hearing impairment.

PTA	N	LTL(mean ± SD)	*P* for trend
1. <25 dBHL (normal)	43	1.270 ± 0.126	**0.03**
2. 26–40 dBHL (mild)	344	1.193 ± 0.092	
3. 41–70 dBHL (moderate)	289	1.189 ± 0.083	
4. >70 dBHL (severe and extremely severe)	33	1.175 ± 0.075	

Limits for the tertiles of T/S ratio were divided as follows: less than 1.155 for the lowest tertile, 1.155–1.219 for the middle tertile, and greater than 1.219 for the highest tertile. Subjects in the highest tertile of LTL were at less risk of developing ARHI than those in the lowest and middle tertiles (adjusted OR for ARHI: 0.327, 95% CI 0.170–0.629, *P* = 0.0008).

## Discussion

Telomeres are TTAGGG repetitive nucleotide sequences at the end of the chromosomes. Telomeric repetitive DNA sequence is synthesized by telomerase via compensating the attrition of the telomeric DNA resulting from cell division. However, telomerase activity is regulated critically as most mature cells scarely express telomerase, except for germline cells, stem cells, and lymphocytes^13^. The variation of telomere length among individuals can be explained by genetics^14^, lifestyle^15^, gender and age to a great extent. A lot of studies have resported that leukocyte telomere length was inversely associated with age, therefore LTL was speculated to be a biomarker of cell senescence and further indicate the risk of age-related diseases, such as cardiovascular disease^8^, type 2 diabetes^9^, and Alzheimer’s Disease^16^ etc. Short telomeres have also been considered as one etiology of the degenerative diseases such as Werner syndrome^17^. Telomere length is vulnerable to oxidative stress. von Zglinicki^18^ presented that accumulation of single-strand breaks resulted from oxidative stress was the major cause of telomere shortening in human diploid fibroblasts. As Seidman [4] summarised, the process of aging had relationship with diverse molecular, biochemical and physiological variations such as decreased mitochondrial activity, increased DNA damage, reduced elasticity of cellular membranes and vascular insufficiency etc. In current literature, various hypotheses have been proposed to explain senescence. Three most convincing theories among them are telomerase theory of aging, mitochondrial clock theory of aging and dysdifferentiation hypothesis of aging. ARHI, as the most common sensory deficit in the elderly population all over the world, reflexes one aspect of aging and its pathogenesis can be attributed to aging, mitochondrial dysfunction, oxidative damage and environmental factors etc. ref. 19. Animal experimental evidence^20^ showed that oxidative damage caused by ROS played a causal role in the development of ARHL. Mitochondria are the major source of ROS. Mitochondrially-targeted catalase can reduce oxidative damage in aged cells through converting toxic H_2_O_2_ into H_2_O and O_2_. As Someya^20^ reported, ROS-induced cochlear DNA damage could be reduced by the overexpression of mitochondrially-targeted catalase, thus delayed the onset of ARHI. Oxidative stress participates in both the attrition of telomere length and the process of ARHI. However, until now no research has reported the association between LTL and ARHI. Thus, we hypothesized that LTL shortening was involved in the pathophysiology of ARHI and proved it in this study.

Gender difference in hearing levels at different frequencies is an acknowledged factor of ARHI, so only male subjects were included in this study. We applied an established quantitative PCR-based technique to measure LTL which has been proven to be useful in the investigation of telomere length and its role in the molecular pathophysiology of aging and various diseases. This study for first time presents that decreased LTL is associated with the presence of ARHI. Subjects in the highest LTL tertile had significantly less risk of developing ARHI compared with the middle and lowest tertiles who owned relatively shorter telomere length. There was a decreasing tendency of LTL as the degree of hearing loss aggravated. The OR between ARHI patients and healthy controls is 0.550, which indicated that, the risk of ARHI decreased by 45% as the T/S ratio increased by 0.1 after taking age into account. Our data showed that longer LTL reduced the incidence of ARHI in the FL, 2–4 kHz AD and mSL subgroups but not in the 8D subgroup. This negative result may be due to the small sample size of the 8D subgroup.

Telomere sequence is highly sensitive to the damage of ROS because of the enrichment of GGG triplet. 8-oxodG is a key oxidative product of guanine which can lead to DNA misreplication. Saretzki^21^ suggested that a p53-dependent cell cycle arrest could be triggered by telomere shortening via accumulation of G-rich single stranded DNA fragments. As telomere attrition has the ability to trigger the activation of p53, and p53 can further lead to Bak-mediated mitochondrial apoptosis which may participate in the progression of ARHI, further studies are warranted to verify whether telomere attrition participates in the pathophysiology of ARHI through activating p53 pathway.

One limitation need to be noted in our study. The number of ARHI patients is much larger than the healthy controls. We conducted “proc surveyselect” process to select 7% cases (N = 47) randomly from the 666 ARHI patients. Then a logistic regression model was applied to estimate the association between LTL and the presence of ARHI adjusted for age (47 cases and 43 controls). We repeated the selection 100 times. 84% of them resulted in *P* < 0.05, suggesting that the low ratio of control. vs. case doesn’t impact the conclusion (Supplementary Table 1).

In conclusion, our study provides to our knowledge the first preliminary evidence that LTL is strongly associated with ARHI. This study suggests a new role for LTL as a mechanism in the pathophysiology of ARHI. In our study, a number of potential factors that may exert influence on telomere attrition in the elderly were not included such as medicine-taking status, lifestyles and other detailed health behaviors. Further studies among various ethnics, longitudinal cohorts are needed to vertify these findings.

## Methods

### Study participants and procedures

Subjects for the cases and controls were recruited at the health check-up centers of Xinhua and Renji Hospital affiliated to Shanghai Jiao Tong University School of Medicine from July 2011 to December 2012. Health check-up centers provide the elderly with annual routine examinations including electrocardiography, chest X-ray and blood biochemical tests etc. As to hearing loss history, noise and ototoxic drug exposure information was collected. Then, an otoscopic examination was performed for each individual to exclude any ear pathology potentially affecting hearing. Clinically, the golden standard for measuring hearing impairment is pure-tone audiometry^22^. Thus, we measured the air and bone conduction thresholds of pure tones by the use of an audiometer (TDH39 earphone, Madsen Itera Inc, Taastrup,DENMARK) in a quiet room. Air and bone conduction thresholds were assessed at 0.25, 0.5, 1, 2, 4, 6, 8 kHz and 0.5, 1, 2, 4 kHz respectively. Diagnostic criteria of ARHL have been presented in our previous study^23^. Subjects with PTA of 0.5 kHz, 1 kHz, 2 kHz, and 4 kHz more than 25 dB in both ears were categorized as the ARHI cases, while those with PTA less than 25 dB in both ears were categorized as the controls. Blood sample was collected from each participant at the time of the physical examination. Informed consent was obtained from each subject. Ethics approval was obtained by the Ethics Committees of both Xinhua Hospital and Renji Hospital affiliated to Shanghai Jiao Tong University School of Medicine. The methods were carried out in accordance with the relevant guidelines, including any relevant details.

### Leukocyte telomere length (LTL) measurement

According to a series of standard procedures, genomic DNA was isolated from peripheral blood leukocytes (DNeasy Blood and Tissue Kit; Qiagen). The extracted DNA was quantified on a Nanodrop 2000 (Thermo Scientific) and was stored at −80 °C until the time of assay. An established and validated quantitative polymerase chain reaction (qPCR) technique was adopted for the telomere length measurement^24^. Relative telomere length for each sample was calculated as the telomere repeat copy number [T/S] ratio using *36B4* (single copy gene) as the reference gene. This strategy aimed to measure the difference between the experimental sample and a reference DNA sample in its ratio of telomere repeat copy number to single copy gene copy number. Thus T/S = 1 when the unknown DNA is equal to the reference DNA in its ratio of telomere repeat copy number to single copy gene copy number. In our study, all the experimental samples were diluted to the same concentration 5 ng/μl. Telomere (T) PCRs and single copy gene (S) PCRs were performed in duplicate for each sample on an ABI StepOnePlus Real-Time PCR System (Applied Biosystems) with 15 ng DNA as a template. Each 20 μl PCR reaction contained 10 μl QuantiNova SYBR Green master mix (2×) (Qiagen) and primers for telomere length measurement^25^: 140 nM Tel-F, 5′-CGGTTTGTTTGGGTTTGGGTTTGGGTTTGGGTTTGGGTT-3′; 140 nM Tel-R, 5′-GGCTTGCCTTACCCTTACCCTTACCCTTACCCTTACCCT-3′ or primers for determinations of the single copy gene *36B4* ^24^: 140 nM 36B4u: 5′-CAGCAAGTGGGAAGGTGTAATCC-3′;140 nM 36B4d: 5′-CCCATTCTATC-ATCAACGGGTACAA-3′. The thermal cycling profile for both telomere and *36B4* started with a 95 °C incubation for 2 minutes, followed by 40 cycles of 5-seconds denaturation at 95 °C and 1-minute annealing/extension at 60 °C. Serially diluted reference DNA standards ranging from 1.25 to 40 ng/μl (2-fold dilution; six data points) were used to generate standard curves respectively for telomere and *36B4* assays on each 96-well plate and all standard curves had a good linearity (R^2^ > 0.99). All the experiments were conducted by laboratory personnel blinded to the case-control classification. The intra- and inter-assay coefficient of variations (CVs) were 1.2% and 6.5% respectively for the telomere length measurement.

### Statistical analysis

Kolmogorov–Smirnov normality test was performed to examine the distribution of T/S ratio and age. Linear regression model was employed to assess the correlation between LTL and age. According to the classification system designed by Cheng-Yung Lee^26^, K-means cluster analysis was used to categorize audiometric shapes in SPSS (version 22) after calibrating the audiometric data to zero dB at 0.25 kHz for baseline homogeneity. It is a stepwise fashion to observe the exact number of audiogram shapes. Clustered results were tested by ANOVA. Optimal end-point should be a maximal F and minimal mean square errors which can explain the heterogeneity among the final number of categorized audiogram shapes and the homogeneity within a specific clustered shape best. Then an age adjusted logistic regression model was used to estimate the association between LTL and ARHI when taking T/S ratio as a continuous variable and ARHI as a binary variable (cases vs controls). A trend analysis was conducted to determine whether LTL was shortened with the hearing impairment worsening. Then, T/S ratio of LTL was divided into three sections among all the subjects: the lowest, middle and highest tertile. Odds ratios (ORs) and 95% confidence intervals (CIs) for the risk of ARHI for the highest tertile compared with the lowest and middle tertiles were calculated with the age adjusted logistic regression model. All reported probability values were two-tailed and the criterion for significance was set at *P* = 0.05. Statistical analysis was performed with SAS software, version 9.2.

## Electronic supplementary material

Supplementary Table 1
