# Supplementary material for: Association of leukocyte telomere length and the risk of age-related hearing impairment in Chinese Hans
Source: Sci Rep. 2017 Aug 31;7:10106. doi: 10.1038/s41598-017-10680-9 (PMC5578975; doi:10.1038/s41598-017-10680-9)
Supplement: Supplementary file 1 — Supplementary Table 1 [file 41598_2017_10680_MOESM1_ESM.pdf]

Association of leukocyte telomere length and the risk of age-related hearing impairment in Chinese Hans

Han Liu, Huajie Luo, Tao Yang, Hao Wu, Dan Chen

Supplementary Table

Supplementary Table 1. Association between T/S ratio and the presence of ARHI (47 cases vs. 43 controls).

"Proc surveyselect" process was conducted to select cases randomly from the 666 ARHI patients (N=47). OR (odds ratio), 95% CI (Confidence Interval) and *P* value indicated the association between T/S ratio of leukocyte telomere length (LTL) and the presence of ARHI by the use of an age adjusted logistic regression model. The selection was repeated 100 times. Results showed that 84% of them resulted in *P*<0.05, suggesting that longer LTL reduced the incidence of ARHI.

	OR adjusted for age	95%CI	*P*
1	0.584	(0.371, 0.918)	0.0199
2	0.507	(0.293, 0.878)	0.0154
3	0.383	(0.199, 0.738)	0.0041
4	0.388	(0.212, 0.712)	0.0022
5	0.513	(0.295, 0.894)	0.0184
6	0.851	(0.525, 1.380)	0.5131
7	0.494	(0.281, 0.870)	0.0146
8	0.527	(0.299, 0.927)	0.0263
9	0.440	(0.235, 0.825)	0.0104
10	0.367	(0.196, 0.688)	0.0018
11	0.480	(0.279, 0.825)	0.0079
12	0.506	(0.286, 0.896)	0.0194
13	0.584	(0.351, 0.972)	0.0384
14	0.560	(0.323, 0.972)	0.0393
15	0.565	(0.330, 0.968)	0.0377
16	0.279	(0.133, 0.584)	0.0007

17	0.649	(0.395, 1.067)	0.0882
18	0.405	(0.213, 0.771)	0.0059
19	0.606	(0.384, 0.954)	0.0304
20	0.358	(0.188, 0.680)	0.0017
21	0.613	(0.402, 0.934)	0.0229
22	0.343	(0.169, 0.694)	0.0029
23	0.627	(0.388, 1.013)	0.0565
24	0.484	(0.264, 0.888)	0.0192
25	0.809	(0.584, 1.120)	0.2022
26	0.624	(0.409, 0.953)	0.0291
27	0.623	(0.399, 0.973)	0.0375
28	0.679	(0.438, 1.053)	0.0840
29	0.494	(0.287, 0.848)	0.0106
30	0.435	(0.246, 0.769)	0.0042
31	0.549	(0.319, 0.947)	0.0310
32	0.409	(0.212, 0.791)	0.0079
33	0.650	(0.434, 0.975)	0.0372
34	0.481	(0.275, 0.841)	0.0102
35	0.421	(0.225, 0.790)	0.0070
36	0.349	(0.180, 0.679)	0.0019
37	0.486	(0.279, 0.845)	0.0107
38	0.611	(0.385, 0.970)	0.0365
39	0.643	(0.388, 1.067)	0.0876
40	0.520	(0.298, 0.909)	0.0217
41	0.476	(0.272, 0.833)	0.0094
42	0.365	(0.191, 0.698)	0.0023
43	0.583	(0.374, 0.910)	0.0175
44	0.499	(0.292, 0.852)	0.0108
45	0.461	(0.275, 0.775)	0.0034

46	0.437	(0.235, 0.812)	0.0088
47	0.649	(0.388, 1.085)	0.0992
48	0.427	(0.229, 0.798)	0.0077
49	0.606	(0.355, 1.033)	0.0655
50	0.371	(0.198, 0.692)	0.0019
51	0.423	(0.230, 0.779)	0.0057
52	0.306	(0.151, 0.619)	0.0010
53	0.345	(0.183, 0.650)	0.0010
54	0.270	(0.132, 0.554)	0.0004
55	0.442	(0.238, 0.822)	0.0099
56	0.492	(0.285, 0.848)	0.0107
57	0.637	(0.381, 1.066)	0.0859
58	0.441	(0.243, 0.802)	0.0073
59	0.587	(0.369, 0.933)	0.0243
60	0.464	(0.268, 0.802)	0.0060
61	0.466	(0.255, 0.852)	0.0132
62	0.605	(0.354, 1.034)	0.0661
63	0.467	(0.241, 0.901)	0.0233
64	0.358	(0.185, 0.693)	0.0023
65	0.726	(0.469, 1.121)	0.1485
66	0.742	(0.474, 1.161)	0.1910
67	0.448	(0.253, 0.795)	0.0061
68	0.372	(0.187, 0.742)	0.0050
69	0.573	(0.345, 0.953)	0.0318
70	0.498	(0.276, 0.897)	0.0202
71	0.608	(0.365, 1.013)	0.0560
72	0.491	(0.278, 0.866)	0.0140
73	0.413	(0.220, 0.775)	0.0059
74	0.558	(0.326, 0.955)	0.0333

75	0.374	(0.190, 0.734)	0.0043
76	0.613	(0.377, 0.995)	0.0478
77	0.470	(0.250, 0.881)	0.0186
78	0.499	(0.284, 0.876)	0.0155
79	0.355	(0.192, 0.657)	0.0010
80	0.578	(0.344, 0.970)	0.0381
81	0.513	(0.293, 0.898)	0.0196
82	0.399	(0.214, 0.744)	0.0038
83	0.532	(0.313, 0.905)	0.0199
84	0.690	(0.438, 1.087)	0.1091
85	0.414	(0.216, 0.792)	0.0077
86	0.658	(0.393, 1.103)	0.1121
87	0.391	(0.207, 0.738)	0.0038
88	0.343	(0.173, 0.678)	0.0021
89	0.604	(0.390, 0.934)	0.0233
90	0.410	(0.215, 0.780)	0.0066
91	0.376	(0.201, 0.704)	0.0022
92	0.323	(0.155, 0.672)	0.0025
93	0.596	(0.368, 0.967)	0.0359
94	0.349	(0.182, 0.670)	0.0016
95	0.509	(0.307, 0.846)	0.0091
96	0.655	(0.419, 1.023)	0.0626
97	0.392	(0.212, 0.726)	0.0029
98	0.269	(0.134, 0.541)	0.0002
99	0.473	(0.266, 0.840)	0.0106
100	0.374	(0.199, 0.703)	0.0022
